# Supplementary material for: Resolving the full spectrum of human genome variation using Linked-Reads
Source: Genome Res. 2019 Apr;29(4):635–45. doi: 10.1101/gr.234443.118 (PMC6442396; doi:10.1101/gr.234443.118)
Supplement: Supplemental Material [file supp_gr.234443.118_Supplemental_longranger-2.2.zip › longranger-2.2.tar.bz2!/lr2.2-public/lib/go/src/code.google.com/vitess/docs/user-guide/reparenting.html]

Vitess / Reparenting


Toggle navigation

- Overview
- Guides
- Reference
- Blog
- GitHub

- #### Overview

  - What is Vitess
  - Scaling MySQL with Vitess
  - Key Concepts
- #### Getting Started

  - Run Vitess on Kubernetes
    - Custom Docker Build
  - Run Vitess Locally
- #### User Guide

  - Introduction- Client Libraries- Backing Up Data- Reparenting
        - Schema Management
        - VSchema Guide
        - Schema Swap (Tutorial)
        - Sharding
          - Horizontal Sharding (Tutorial, automated)
          - Horizontal Sharding (Tutorial, manual)
          - Sharding in Kubernetes (Tutorial, automated)
          - Sharding in Kubernetes (Tutorial, manual)
        - Vitess Sequences
        - MySQL Server Protocol
        - Vitess and Replication
        - Update Stream
        - Row Based Replication
        - Topology Service
        - Transport Security Model
        - Launching
          - Scalability Philosophy
          - Production Planning
          - Server Configuration
          - 2PC Guide
          - Troubleshooting
        - Upgrading
- #### Reference Guides

  - Vitess API- vtctl Commands
- #### Other Resources

  - Presentations- Blog- Roadmap
- #### Contributing

  - Contributing to Vitess- GitHub Workflow- Code Reviews
- GitHub

# Reparenting

- #### Overview

  - What is Vitess
  - Scaling MySQL with Vitess
  - Key Concepts
- #### Getting Started

  - Run Vitess on Kubernetes
    - Custom Docker Build
  - Run Vitess Locally
- #### User Guide

  - Introduction- Client Libraries- Backing Up Data- Reparenting
        - Schema Management
        - VSchema Guide
        - Schema Swap (Tutorial)
        - Sharding
          - Horizontal Sharding (Tutorial, automated)
          - Horizontal Sharding (Tutorial, manual)
          - Sharding in Kubernetes (Tutorial, automated)
          - Sharding in Kubernetes (Tutorial, manual)
        - Vitess Sequences
        - MySQL Server Protocol
        - Vitess and Replication
        - Update Stream
        - Row Based Replication
        - Topology Service
        - Transport Security Model
        - Launching
          - Scalability Philosophy
          - Production Planning
          - Server Configuration
          - 2PC Guide
          - Troubleshooting
        - Upgrading
- #### Reference Guides

  - Vitess API- vtctl Commands
- #### Other Resources

  - Presentations- Blog- Roadmap
- #### Contributing

  - Contributing to Vitess- GitHub Workflow- Code Reviews

**Reparenting** is the process of changing a shard's master tablet
from one host to another or changing a slave tablet to have a
different master. Reparenting can be initiated manually
or it can occur automatically in response to particular database
conditions. As examples, you might reparent a shard or tablet during
a maintenance exercise or automatically trigger reparenting when
a master tablet dies.

This document explains the types of reparenting that Vitess supports:

- *Active reparenting* occurs when the Vitess
  toolchain manages the entire reparenting process.
- *External reparenting* occurs when another tool
  handles the reparenting process, and the Vitess toolchain just updates its
  topology server, replication graph, and serving graph to accurately reflect
  master-slave relationships.

**Note:** The `InitShardMaster` command defines the initial
parenting relationships within a shard. That command makes the specified
tablet the master and makes the other tablets in the shard slaves that
replicate from that master.

## MySQL requirements

Vitess supports MySQL 5.6, MySQL 5.7 and MariaDB 10.0 implementations.

### GTIDs

Vitess requires the use of global transaction identifiers
(GTIDs) for its operations:

- During active reparenting, Vitess uses GTIDs to initialize the
  replication process and then depends on the GTID stream to be
  correct when reparenting. (During external reparenting, Vitess
  assumes the external tool manages the replication process.)
- During resharding, Vitess uses GTIDs for
  filtered replication,
  the process by which source tablet data is transferred to the proper
  destination tablets.

### Semisynchronous replication

Vitess does not depend on
semisynchronous replication but does work if it is implemented.
Larger Vitess deployments typically do implement semisynchronous replication.

## Active Reparenting

You can use the following `vtctl`
commands to perform reparenting operations:

- `PlannedReparentShard`
- `EmergencyReparentShard`

Both commands lock the shard for write operations. The two commands
cannot run in parallel, nor can either command run in parallel with the
`InitShardMaster`
command.

The two commands are both dependent on the global topology server being
available, and they both insert rows in the topology server's
`_vt.reparent_journal` table. As such, you can review
your database's reparenting history by inspecting that table.

### PlannedReparentShard: Planned reparenting

The `PlannedReparentShard` command reparents a healthy master
tablet to a new master. The current and new master must both be up and
running.

This command performs the following actions:

1. Puts the current master tablet in read-only mode.
2. Shuts down the current master's query service, which is the part of
   the system that handles user SQL queries. At this point, Vitess does
   not handle any user SQL queries until the new master is configured
   and can be used a few seconds later.
3. Retrieves the current master's replication position.
4. Instructs the master-elect tablet to wait for replication data and
   then begin functioning as the new master after that data is fully
   transferred.
5. Ensures replication is functioning properly via the following steps:
   1. On the master-elect tablet, insert an entry in a test table
      and then update the global `Shard` object's
      `MasterAlias` record.
   2. In parallel on each slave, including the old master, set the new
      master and wait for the test entry to replicate to the slave tablet.
      (Slave tablets that had not been replicating before the command was
      called are left in their current state and do not start replication
      after the reparenting process.)
   3. Start replication on the old master tablet so it catches up to the
      new master.

In this scenario, the old master's tablet type transitions to
`spare`. If health checking is enabled on the old master,
it will likely rejoin the cluster as a replica on the next health
check. To enable health checking, set the
`target_tablet_type` parameter when starting a tablet.
That parameter indicates what type of tablet that tablet tries to be
when healthy. When it is not healthy, the tablet type changes to
`spare`.

### EmergencyReparentShard: Emergency reparenting

The `EmergencyReparentShard` command is used to force
a reparent to a new master when the current master is unavailable.
The command assumes that data cannot be retrieved from the current
master because it is dead or not working properly.

As such, this command does not rely on the current master at all
to replicate data to the new master. Instead, it makes sure that
the master-elect is the most advanced in replication within all
of the available slaves.

**Important:** Before calling this command, you must first identify
the slave with the most advanced replication position as that slave
must be designated as the new master. You can use the
`vtctl ShardReplicationPositions`
command to determine the current replication positions of a shard's slaves.

This command performs the following actions:

1. Determines the current replication position on all of the slave
   tablets and confirms that the master-elect tablet has the most
   advanced replication position.
2. Promotes the master-elect tablet to be the new master. In addition to
   changing its tablet type to `master`, the master-elect
   performs any other changes that might be required for its new state.
3. Ensures replication is functioning properly via the following steps:
   1. On the master-elect tablet, Vitess inserts an entry in a test table
      and then updates the `MasterAlias` record of the global
      `Shard` object.
   2. In parallel on each slave, excluding the old master, Vitess sets the
      master and waits for the test entry to replicate to the slave tablet.
      (Slave tablets that had not been replicating before the command was
      called are left in their current state and do not start replication
      after the reparenting process.)

## External Reparenting

External reparenting occurs when another tool handles the process
of changing a shard's master tablet. After that occurs, the tool
needs to call the
`vtctl TabletExternallyReparented`
command to ensure that the topology server, replication graph, and serving
graph are updated accordingly.

That command performs the following operations:

1. Locks the shard in the global topology server.
2. Reads the `Shard` object from the global topology server.
3. Reads all of the tablets in the replication graph for the shard.
   Vitess does allow partial reads in this step, which means that Vitess
   will proceed even if a data center is down as long as the data center
   containing the new master is available.
4. Ensures that the new master's state is updated correctly and that the
   new master is not a MySQL slave of another server. It runs the MySQL
   `show slave status` command, ultimately aiming to confirm
   that the MySQL `reset slave` command already executed on
   the tablet.
5. Updates, for each slave, the topology server record and replication
   graph to reflect the new master. If the old master does not return
   successfully in this step, Vitess changes its tablet type to
   `spare` to ensure that it does not interfere with ongoing
   operations.
6. Updates the `Shard` object to specify the new master.

The `TabletExternallyReparented` command fails in the following
cases:

- The global topology server is not available for locking and
  modification. In that case, the operation fails completely.

Active reparenting might be a dangerous practice in any system
that depends on external reparents. You can disable active reparents
by starting `vtctld` with the
`--disable_active_reparents` flag set to `true`.
(You cannot set the flag after `vtctld` is started.)

## Fixing Replication

A tablet can be orphaned after a reparenting if it is unavailable
when the reparent operation is running but then recovers later on.
In that case, you can manually reset the tablet's master to the
current shard master using the
`vtctl ReparentTablet`
command. You can then restart replication on the tablet if it was stopped
by calling the `vtctl StartSlave`
command.

Contact: vitess@googlegroups.com  **·**  
Announcements  **·**  
© 2017 Vitess powered by Google Inc
